# Virtual microscopy system at Chinese medical university: an assisted teaching platform for promoting active learning and problem-solving skills

**DOI:** 10.1186/1472-6920-14-74

**Published:** 2014-04-09

**Authors:** Yanping Tian, Wengang Xiao, Chengren Li, Yunlai Liu, Maolin Qin, Yi Wu, Lan Xiao, Hongli Li

**Affiliations:** 1 Department of Histology and embryology, Third Military Medical University, 30# Gaotanyan St, Chongqing, Shapingba District 400038, China; 2 College of Basic Medical Sciences, Third Military Medical University, Chongqing 400038, China

**Keywords:** Virtual microscope, Active learning, Problem-solving skills, Assisted platform, Chinese medical university

## Abstract

**Background:**

Chinese medical universities typically have a high number of students, a shortage of teachers and limited equipment, and as such histology courses have been taught using traditional lecture-based formats, with textbooks and conventional microscopy. This method, however, has reduced creativity and problem-solving skills training in the curriculum. The virtual microscope (VM) system has been shown to be an effective and efficient educational strategy. The present study aims to describe a VM system for undergraduates and to evaluate the effects of promoting active learning and problem-solving skills.

**Methods:**

Two hundred and twenty-nine second-year undergraduate students in the Third Military Medical University were divided into two groups. The VM group contained 115 students and was taught using the VM system. The light microscope (LM) group consisted of 114 students and was taught using the LM system. Post-teaching performances were assessed by multiple-choice questions, short essay questions, case analysis questions and the identification of structure of tissue. Students’ teaching preferences and satisfaction were assessed using questionnaires.

**Results:**

Test scores in the VM group showed a significant improvement compared with those in the LM group (p < 0.05). There were no substantial differences between the two groups in the mean score rate of multiple-choice questions and the short essay category (p > 0.05); however, there were notable differences in the mean score rate of case analysis questions and identification of structure of tissue (p < 0.05). The questionnaire results indicate that the VM system improves students’ productivity and promotes learning efficiency. Furthermore, students reported other positive effects of the VM system in terms of additional learning resources, critical thinking, ease of communication and confidence.

**Conclusions:**

The VM system is an effective tool at Chinese medical university to promote undergraduates’ active learning and problem-solving skills as an assisted teaching platform.

## Background

Because of China’s vast population, there is a shortage of doctors. As a result there has been a significant increase in the number of students entering medical school. China’s medical education system is the largest in the world; in 2008 there were 159 medical teaching institutions and approximately 1.7 million students [1]. Because of the high number of students, shortage of teachers and limited equipment, Chinese teaching usually relies on rote learning and instruction instead of active investigation by students, which can promote creativity and imagination [2]. Compared with most developed countries in Europe and North America, China faces some unique problems within its medical education as a result of its history and culture [2,3].

Education and training in histology, anatomical pathology and cytopathology remain essential to both undergraduate and postgraduate students. At most universities in China, histology courses are traditionally lecture-based, incorporating the use of textbooks, glass slides and conventional microscopy. These courses have been confronted by resource limitations, including too many students and not enough space to accommodate them all, and very few instructors and teaching resources, including human specimens and microsections [3]. Circumstances such as these can act to reduce creativity and problem-solving skills training in the curriculum. To enhance these skills, schools in North America and Europe currently have used problem-based learning (PBL), case-reinforced learning (CRL) education methodologies and new e-based technologies and approaches [3]. The PBL methodology, which aims to cultivate students’ creativity and practical abilities, has been used in China since the mid-1980s. While some recent studies have shown that analytical and problem-solving skills have improved among PBL students, the application of PBL is limited because of faculty shortage and a lack of resources [4]. However, strategies to enhance students’ active learning and problem-solving skills are increasingly used in the better-known Chinese medical schools.

Over the last two decades, the number of web-based histology resources has expanded dramatically, with centralized histological resources being simultaneously provided to many students. Virtual microscope (VM) is a computer-based program that enables viewing, navigating and annotating of digital slides, and is similar to working with a real microscope [5,6]. The VM system for basic medical education and quality control overcomes several traditional problems, such as faculty shortages and lack of resources [7-9]. There are some published studies addressing VM in several countries, particularly in North America and Europe [10]; however, little comparative information on education and professional development is available in developing countries, especially China. New teaching methodologies are required for Chinese students to ensure that the purpose of the current educational system is achieved, and to improve active learning and problem-solving skills [4,11].

This project aims to describe and discuss the VM system for Chinese undergraduate histology courses, and explores the outcomes of the VM system with the hope that histology teaching will become more process-oriented. We designed our VM system as an open assisted platform to empower our learners to develop active learning and problem-solving skills to overcome the limitations facing traditional lecture-based learning.

## Methods

### Participating students

The participating students were 229 second-year students at the Third Military Medical University. These students enrolled in the course directly from high school in the autumn of 2011. Their first year consisted of a basic science curriculum, which includes mathematics, chemistry, physics and anatomy. The students then began their basic medical curriculum in the third semester, which includes histology courses. Based on the students’ academic record from their first academic year, the students were assigned into two groups. Group 1 was scheduled for virtual microscopes and thus became the VM group, and Group 2 was scheduled for light microscopes, thus becoming the LM group. A pre-test was conducted before the course and the results showed no difference between the two groups. The teaching staff comprised six teachers experienced in teaching histology. Table 1 shows the basic statistical data of both groups: VM group and LM group.

**Table 1:** Basic statistical data of VM group and LM group

**Groups**	**VM group (n = 115)**	**LM group (n = 114)**
Male/Female	101/14	100/14
Mean age (years)	19.2	19.4

### VM system of our university

**Figure 1:**
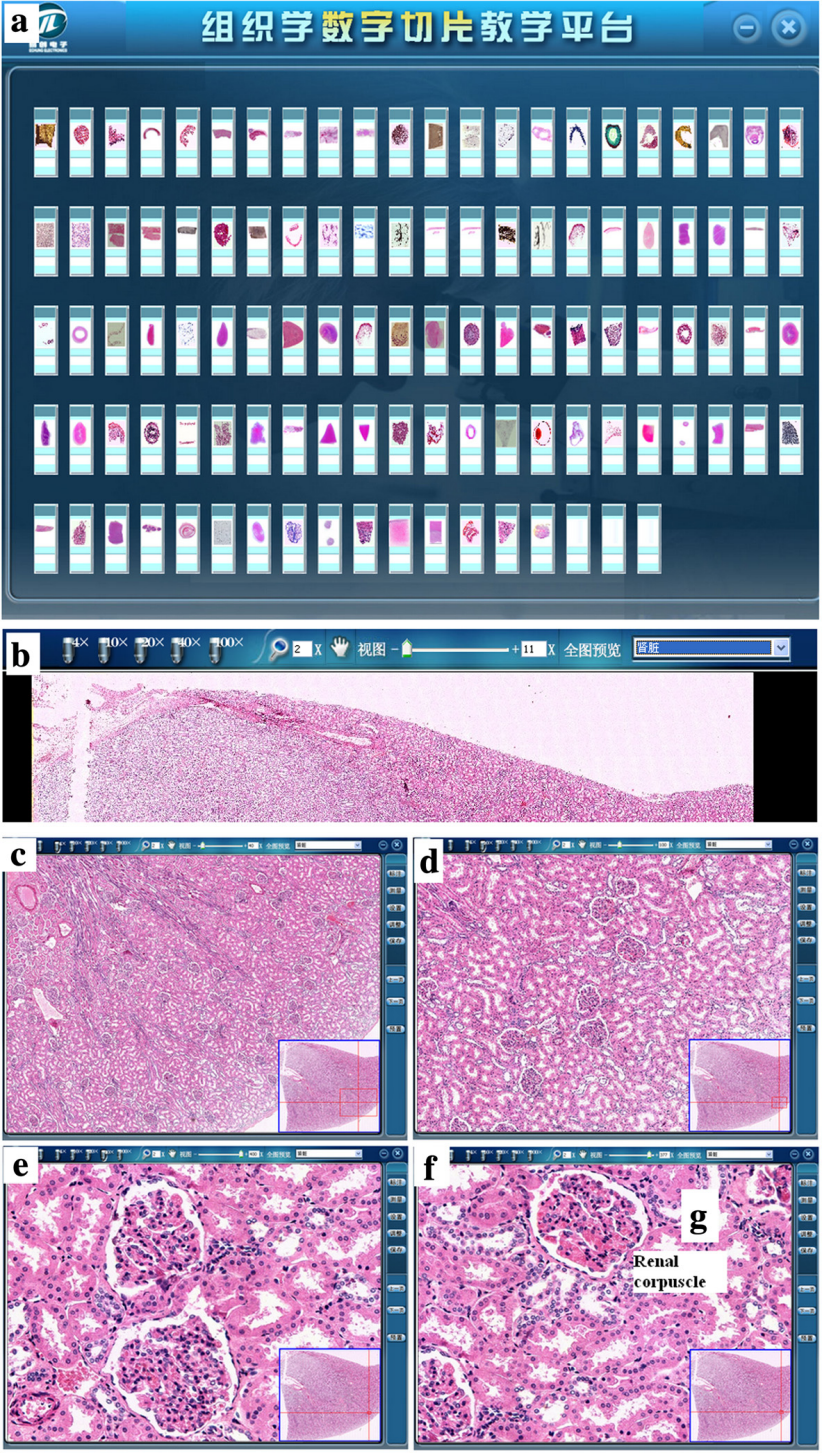
**Screen captures of the interface for histology teaching program at TMMU.** The top screen shows the navigation tool that uses a Palm Pilot as a metaphor to navigate through the content of the program. The ‘virtual slides’ can be viewed from personal computers with a web browser **(a)**. The window consists of a main viewing area with a mini-map to provide a navigational overview. The user can use the toolbar at the top, including 4×, 10×, 20×, 40×, 100× and X × **(b)**, to manipulate the image on the computer screen by zooming in and out of the virtual slide displayed in the upper-left-hand corner; the user can adjust for contrast and brightness in the right-hand corner **(c**–**f)**. Regions of special interest (ROI) were indicated and some annotations added **(g)**. The possibility to determine the size of an object is helpful, especially in determining the size of entire specimens.

The Echung Electronics Company helped us establish a local network and provided the technical support for our VM system. The entire collection of glass slides was scanned and photographed at high magnification. The slide-viewing screen is shown in Figure 1. The generated ‘virtual slides’ were uploaded to a database server for web-based viewing (Figure 1a). The window consists of a main viewing area with a mini-map to provide a navigational overview. Users can zoom into the image on the screen using the toolbar (4×, 10×, 20×, 40×, 100× and X × (Figure 1b)) in the upper-left-hand corner. The user can also adjust for contrast and brightness using the button in the right-hand corner (Figure 1c–f). There is also a button that can mark certain areas and save user comments regarding those areas (‘annotations’). Clicking on the marker title in the sidebar will center the marker on the map and magnify the image to the level chosen by the user when creating that annotation (Figure 1g). The mini-map can be collapsed; this enables almost the entire screen to be used for slide viewing. Broadcast teaching, online cinema and operations were also applied in the system. Furthermore, advanced features, including a search function, site usage tracking and the listing of all used slides, were made possible with the database structure. Via e-mail and discussion boards, the students expressed any concerns to teachers and other students, which increased the amount of mutual reflection.

### Teaching methods

VM group: the descriptive data of the group are displayed in Table 1. The group of 115 students was randomly divided into 12 small groups, each composed of 9 to 10 participants. At the beginning of the class, clinical case materials and the questions shown in Table 2 were transmitted via computers. Students viewed slides through the VM system based on the materials and questions. They were also allowed to view glass slides by LM. All materials/slides and learning issues, as well as the discussion notes, were organized into rubrics using an online course management system. Individual group discussions were not supervised and were subsequently followed by an open classroom discussion with all groups participating. During the open class discussion, the teacher took detailed notes on the blackboards. The instructor used guiding questions to ensure that students identified learning issues that were appropriate to the case and consistent with the learning objectives of the course. The case was concluded with a discussion of the final hypotheses using a random reporting format.

**Table 2:** Clinical materials of a case and guiding questions

**Case**	**Questions**
A 41 years old male hypertensive patients to the hospital examination revealed hematuria and proteinuria, angiotensin concentration increased, edema, injection of vasopressin, edema disappeared slowly.	A. Considering from the urinary system, what is the cause of high blood pressure? What is the organizational structure?
	B. Which structure of tissue was damaged resulting in proteinuria?
	C. which structure did vasopressin hormone affect?

LM Group: the descriptive data of the group are displayed in Table 1. The 114 students underwent traditional lecture-based teaching, provided by the same staff as the VM group. Students observed equivalent glass slides by LM as those in the VM group. There were also open classroom discussions but no clinical case materials.

### Evaluation methods

Three different approaches were used to evaluate the study.

– 1. Written histology examination at the end of the semester: the students in both groups took the final examination after finishing the course. The examination was designed as a combined multiple-choice, short essay and case analysis test: 100 multiple-choice questions (0.5 points per question), 10 short essay questions (3 points per question) and 2 case analysis questions (10 points per question). Standard answers for all questions were defined and handed out to the staff before the students’ examination answers were scored. The scoring staff were blind to the identity of the students and to their assigned group.

– 2. The exam on tissue structure at the end of the semester included the identification of tissue structure: 15 whole organ sections were fixed and stained on glass slides (hematoxylin and eosin (HE) staining). Students observed the slides by optical microscope and identified the structures of organs (1 point per organ).

– 3. A questionnaire was given to determine students’ thoughts about the VM system: both the VM group and the LM group filled out this questionnaire to rate the content, framework and subjective effects of the VM course. The questionnaires consisted of 25 questions. Answers were provided on five-point Likert scales, ranging from 1, ‘strongly disagree,’ to 5, ‘strongly agree.’ In addition, students had the opportunity to comment on the content of the tutorial.

### Ethical approval

This study was approved by the Protocol Review Committee of the Undergraduate MD Programme and the Faculty of Health Sciences Research Ethics Board at Third Military Medical University.

### Statistical analyses

The data from the students’ evaluation ratings were summarized using descriptive statistics (means, standard deviation and response rates). Statistical analysis was conducted using the software SPSS 17.0 for Windows (SPSS Inc., Chicago, IL, USA). Data are presented as means ± SD. Statistical analysis between groups was evaluated using T-tests and analysis of variance (ANOVA); p-value < 0.05 was considered significant.

## Results

### Participation

**Figure 2:**
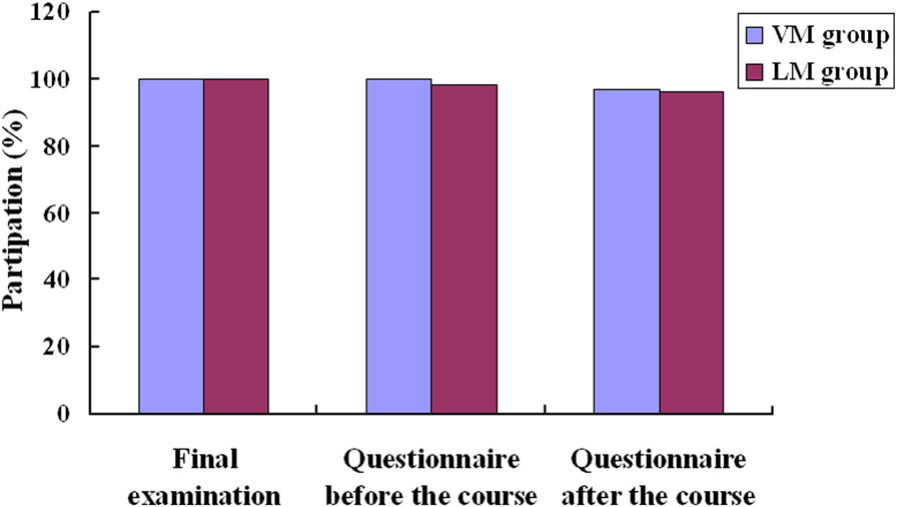
Participation rates in completing the final examination and questionnaires on preference for VM or LM before and after the course.

As shown in Figure 2, all 229 students originally included in the study took the histology final examination: 115 students in the VM group and 114 in the LM group. Before the course, 100% of students in the VM group and 98% in the LM group completed the questionnaire on their preference for VM or LM; after the course, the completion rates were 97% and 96%, respectively.

### Histology examination results of the two groups

**Figure 3:**
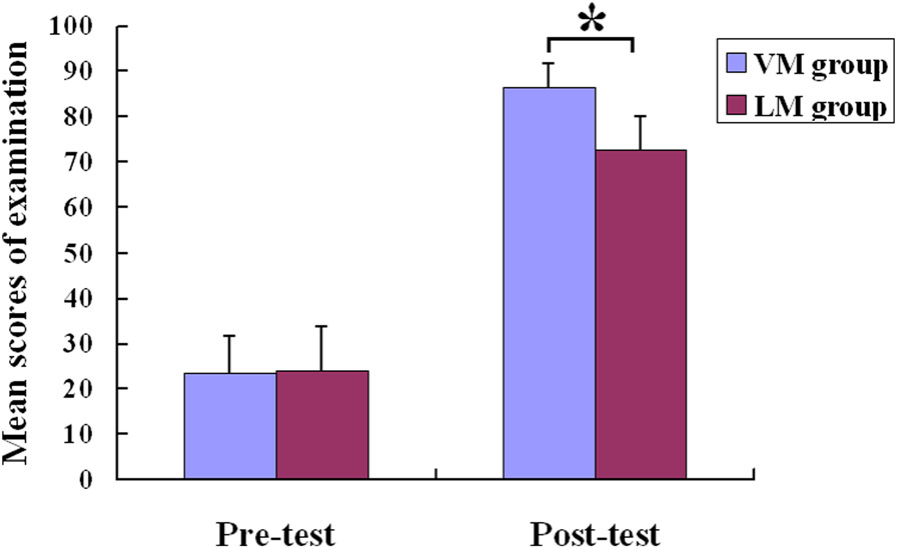
**Mean examination scores (X ± SD) for the pre**- **and post**-**tests in the final examination of histology: VM group (n = 115) and LM group (n = 114).** *p < 0.05.

**Figure 4:**
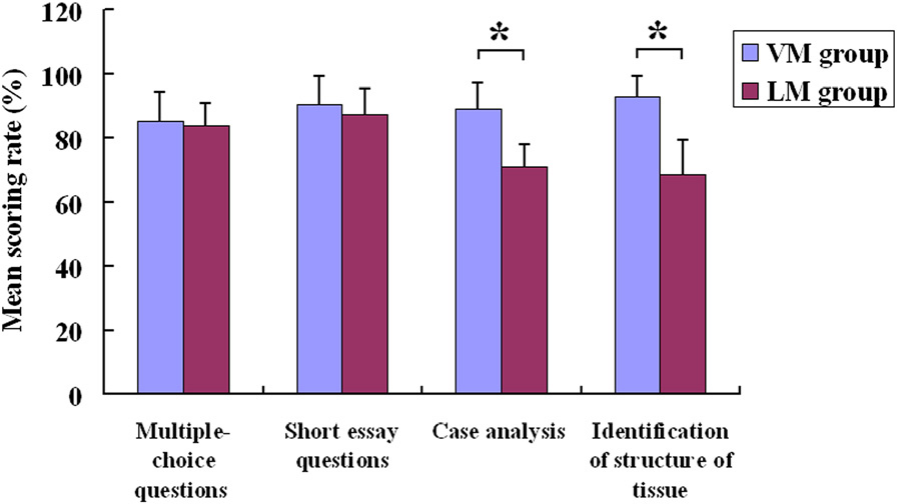
**Comparison of the two groups’ results in multiple-choice questions, short essay and case analysis, respectively.** *p < 0.05.

To determine the students’ knowledge gain, a pre-test before the course and a post-test after the course was organized and the differences between test scores were analyzed. The results showed no difference among the students’ pre-test scores prior to the course, but post-test results displayed a significant difference between the results of the VM group (mean ± SD, 85.6 ± 9.7) and those of the LM group (71.2 ± 8.4), p < 0.05 (Figure 3). The results of post-test was showed in Figure 4, there were no significant differences between the two groups (85.3% ± 8.9% and 90.4% ± 9.5% in the VM group versus 83.7% ± 7.5% and 87.5% ± 8.7% in the LM group, p > 0.05) in the mean score rate of the multiple-choice questions and the short essay category. However, an analysis of the mean score rate of the case analysis and tissue structure identification sections revealed significant differences (89.1% ± 8.2% and 93.1% ± 6.7% in the VM group versus 70.8% ± 7.1% and 68.5% ± 10.9% in the LM group, p < 0.05).

### Analysis of student questionnaire

After the trial, the 115 students in the VM group were asked to fill out an anonymous, self-structured questionnaire. The students’ profile evaluation results are shown in Table 3. Students declared a high level of satisfaction with the VM system (mean: 4.5 on the five-point Likert-scale). The functionality of the VM system was evaluated. As shown in Table 3, the VM courses were viewed as efficient in terms of undergraduate education (4.5). Participants mentioned that the combination of narration and images improved their understanding and retention. They identified class discussions, message board usage and clinical cases as the most useful elements of the VM system. Students believed the VM system not only improved their communication ability (4.2) but also their ability to learn independently, their confidence and their researching ability (4.0). The students considered the VM system to be an effective learning strategy (4.5) and would recommend it to other students (4.3).

**Table 3:** **Mean ratings of medical students**’ **profile questions in VM group using a Five**-**Point Likert**-**style Scale With 1** = **strongly Disagree and 5** = **strongly Agree**

**Survey question**	**Mean ± SD**
1. I am interested in subject of the VM course	4.4 ± 0.5
2. The virtual microscope is facilitated and convenient to learn histology.	4.2 ± 1.0
3. The design of the modules keeps me focused on the subject matter	3.8 ± 1.2
4. The navigation of the slides is easy to select specific content	4.6 ± 0.7
5. The graphics and animations are appropriate and relevant to the content	3.5 ± 1.4
6. The graphics and animations help me in reaching the learning objectives	4.0 ± 0.8
7. The content is sufficient to master the stated learning objectives	4.1 ± 1.1
8. The content is clearly organized and easily understood	3.9 ± 1.4
9. The system accommodate multiple learning styles	4.4 ± 0.9
10. The VM system is convenient to discuss with groups and teachers.	4.6 ± 1.1
11. I felt the system functions (search function, slide list, self-test etc.) added to the interactive atlas throughout the semester made the atlas a better educational tool.	4.5 ± 1.2
12. I felt that the atlas positively affected my learning experience by making structure identification a quicker and less confusing process.	4.3 ± 1.2
13. The modules help in remediating and improving my learning and monitoring the progress of my learning	4.3 ± 1.1
14. The cases have motivated me to use additional learning resources.	4.0 ± 1.2
15. The course increases my motivation to do well in the course.	4.3 ± 1.3
16. The course encourages critical thinking at available material.	4.1 ± 1.3
17. In the VM group, I could improve my communication skills.	4.2 ± 1.0
18. The course improves my ability of active learning, confidence and researching.	4.0 ± 1.5
19. In the VM course I learned something for my dealing with patients.	4.5 ± 0.7
20. The tutor guides the VM course adequately.	3.5 ± 1.4
21. The tutor attends session and control of session for discussion is adequate.	3.7 ± 1.1
22. I consider the VM course to be important within the frame of my studies	4.1 ± 1.3
23. I consider VM course to be an effective learning style for myself.	4.5 ± 1.2
24. I achieve my learning objectives using the VM system.	4.1 ± 0.7
25. I will recommend this VM course to other students.	4.3 ± 0.7

As students in the LM group did not participate in the VM system, they did not respond to the content, framework and subjective effects of the VM course. However, their level of satisfaction with the LM histology course was lower than that of the VM group. They also wished to use the VM system in future histology courses.

## Discussion

One of the key skills that histopathologists need to learn is the ability to identify areas of diagnostic relevance from an entire sample. Our VM system can be exploited to increase students’ basic knowledge and problem-solving skills despite the lack of available space, equipment and instructors in traditional LM settings. This technology is referred to as virtual microscopy and has created enormous opportunities in histological training and education. Using this method, students can learn key histopathological skills, such as identifying areas of diagnostic relevance from an entire slide [12].

Medical curricula are changing to reduce scheduled time for basic science instruction as well as focusing on student-centered learning approaches. Histology laboratory instruction is moving away from the sole use of the traditional combination of LM and glass slides in favor of virtual microscopy and virtual slides [13]. Virtual microscopy represents one such simulation-based technology that has the potential to enhance student learning and readiness to practice while revolutionizing clinical problem-solving skills [10]. Thus, virtual microscopy is a reliable and more reproducible technology and has several advantages over glass slides. The VM system is easily accessible by faculty and students inside and outside the laboratory, overcoming the limitations of space and equipment. Groups of students can view exactly the same specimen, allowing for the same point of reference in group work and discussions. Moreover, the use of annotations on the virtual slides is found to be a useful way for interpreting findings in textbooks [14-17].

As our university is a military medical school, all the students are soldiers and histology is one of their basic medical training requirements; both the VM group and LM group maintained a high participation rate in our study. Because there are more students than available teachers and equipment, traditional Chinese teaching usually relies on a lecture-based format as the formal approach to preclinical training. This rote teaching approach facilitates retention of knowledge but fails to effectively integrate students’ skills of critical thinking and decision-making [2,3]. The VM system can provide an opportunity to learn critically in the setting of limited physical resources. Of the 115 participants who studied histology using the VM system over one semester, 95% preferred to use virtual microscopy. By examining the students’ test scores, we found that their knowledge of histology significantly improved after the course compared with students using LM. Students indicated that the VM helped to stimulate their interest in the topic before the discussion, enhanced their ability to memorize the information and helped them to become more active participants. This result is consistent with previous reports [18].

The structure characterization of tissue is one of the key skills for histopathologists. The VM system enables this skill to be developed in a web-based environment by viewing, navigating, and annotating digital slides, where the student/trainee can see the morphologic patterns or diagnostic feature in the context of the entire sample [5,12]. Questionnaire results showed nearly all the students (99%) found the virtual slides easy to navigate, and 93% thought that the virtual image quality was at least as good as that of a normal microscope. In the VM teaching trials, the students learned to effectively use various sources of information and were trained in the rapid retrieval of relevant clinical pathological knowledge. These are important skills for medical professionals who must act quickly in real clinical problems. Under the advantages of the VM system, the VM students achieved better results in the categories of identification of tissue structure and case analysis questions. Similarly, previous studies have also reported positive attitudes toward the use of virtual slides [19]. However, the students’ results showed no differences in the short essay questions and multiple-choice questions. This indicates that the short essay and multiple-choice questions require more complex levels of knowledge (i.e., regarding comprehension or analysis). This result supports a study by Krippendorf and Lough [20], which demonstrated a significant improvement in the identification of tissue structure and case analysis exam performance by students who learned using virtual microscopy when compared with those who used conventional microscopy.

The VM system also provides variant training opportunities. Virtual microscopy has the benefit of delivering histology courses to students outside the classroom setting. Students can access online virtual microscopy courses at any time and place, allowing them to view slides that would have traditionally been restricted to the slide box and classroom. Thus, students no longer need to be in the same room as the glass slide. The student surveys indicate that the VM enhances student confidence through communication with others and the ability to identify structures by annotation; it improves their productivity, promotes learning efficiency and positively affects their learning experience (Table 3). The VM system was also able to meet the students’ various study preferences [21]. The online educational environment with collaborative assignments and interactive content was a novel experience.

Although it has provided us with a new, convenient and effective teaching method, VM-based education indicates the loss of training in manual traditional light microscopy. This is a controversial topic for many schools who recognize that students may have the opportunity to use LMs in settings outside the course, such as clerkship rotations or research projects [21]. Compared with traditional light microscopy, the VM course is more suited for Chinese undergraduate education; however, the instructional model needs further development to be appropriate for training.

In conclusion, the integration of the digital slide-based virtual microscopy into conventional courses is an important step towards a blended learning strategy. Students can regularly use the VM system and enjoy the opportunity of learning without temporal or spatial restrictions and enhance their active learning and problem-solving skills. We believe that these interactive programs provide a better approach to interactive learning as an assisted platform.

## Conclusions

Through the cooperative efforts of faculty and students, our VM system is an effective solution as an assisted platform against the limitations facing traditional optical laboratories, enabling us to implement new, collaborative teaching and learning strategies. Students can use the VM system regularly and augment their basic knowledge and problem-solving skills.

## Competing interests

The authors declare that they have no competing interests.

## Authors’ contribution

Y T and M Q, MD, are Senior Lecturers. They teach courses and run workshops. C L and Y L, MD are assistant professors. They teach courses and run workshops. W X and Y W work at the College of Basic Medical Sciences, run workshops in medical education and take part in evaluations and research projects in education. L X and H L, MD, professor, have extensive experience in education and educational research. They designed the project. All authors read and approved the final manuscript.

## Pre-publication history

The pre-publication history for this paper can be accessed here:

http://www.biomedcentral.com/1472-6920/14/74/prepub
